# The association between cognitive function trajectories and all-cause mortality in middle-aged and older Chinese adults with cardiovascular disease: A longitudinal study from CHARLS^☆^

**DOI:** 10.1016/j.ijcrp.2026.200583

**Published:** 2026-01-29

**Authors:** Xiaopeng Song, Yufei Wang, Hua Chen

**Affiliations:** a Department of Cardiology, Inner Mongolia Autonomous Region People's Hospital, Hohhot City, China; b Graduate School, Inner Mongolia Medical University, Hohhot City, China

**Keywords:** Cognitive trajectory, Cardiovascular disease, All-cause mortality

## Abstract

**Objective:**

To identify distinct multi-year cognitive function trajectories in middle-aged and older Chinese adults with established cardiovascular disease (CVD) and evaluate their independent associations with all-cause mortality.

**Methods:**

This prospective study utilized data from five waves (2011–2020) of the China Health and Retirement Longitudinal Study (CHARLS). A total of 1464 participants aged ≥45 years with CVD were included. Global cognitive scores (range 0–21) were assessed at three time points (2011, 2013, and 2015). A Longitudinal K-means clustering algorithm with Dynamic Time Warping was employed to identify cognitive trajectories. Cox proportional hazards models were used to estimate hazard ratios (HRs) and 95 % confidence intervals (CIs) for all-cause mortality during a mean follow-up of 4.7 years.

**Results:**

Four distinct cognitive trajectories were identified: High-Baseline-Relatively-Stable (39.1 %), Mid-High-Baseline-Significant-Improvement (31.2 %), Mid-Baseline-Rapid-Decline (16.7 %), and Low-Baseline-Relatively-Stable (13.0 %). Using the Mid-High-Baseline-Significant-Improvement group as the reference, both the Mid-Baseline-Rapid-Decline trajectory (adjusted HR = 2.01; 95 % CI: 1.32–3.05) and the Low-Baseline-Relatively-Stable trajectory (adjusted HR = 1.82; 95 % CI: 1.10–3.00) were significantly associated with an increased risk of all-cause mortality after adjusting for covariates. The association was notably stronger among participants with hypertension (P for interaction = 0.009).

**Conclusions:**

Among middle-aged and older adults with CVD, trajectories characterized by rapid cognitive decline or persistently low function are powerful, independent predictors of all-cause mortality. In contrast, cognitive improvement is not associated with excess mortality risk. These findings underscore the prognostic importance of dynamic cognitive assessment and suggest that monitoring cognitive trajectories may aid in the risk stratification of CVD patients.

## Highlights

• Identified four distinct longitudinal cognitive patterns in CVD patients.
• Rapid cognitive decline and low-stable function predict higher mortality.
• Cognitive improvement was not associated with increased mortality risk.
• Mortality risk from cognitive decline was strongest in hypertensive patients.

## 1 Introduction

Cardiovascular disease (CVD) and cognitive decline are leading causes of morbidity and mortality in aging societies [[1], [2], [3], [4]], with their co-occurrence signifying a particularly adverse prognostic state [5,6]. Although the association between poor baseline cognitive performance and increased mortality risk in CVD patients is established, cognitive function is inherently dynamic.

A recent meta-analysis indicates that cognitive function is strongly associated with CVD prognosis. Among adults and older adults with cognitive impairment, the risk of cardiovascular mortality was 75 % higher compared to those without cognitive impairment [7]. Other studies have examined the relationship between cognitive scores as a continuous variable and the risk of CVD mortality in middle-aged and older adults, consistently demonstrating that lower cognitive function is significantly associated with an increased risk of death from CVD [[8], [9], [10]].

Accurate risk stratification is crucial for the management of elderly patients with CVD. Previous studies have developed robust mortality prediction scores for elderly patients, such as those with heart failure or identifying cardiac variables associated with outcomes in patients with pacemakers [11,12]. However, while incorporating various clinical metrics, current risk stratification largely overlooks the longitudinal dimension of cognitive health.

A shift from static, cross-sectional assessment to the study of cognitive trajectories is necessary to capture critical information about disease progression and resilience. Most prior studies have relied on a single assessment, treating cognitive function as a fixed baseline covariate. This approach fails to distinguish between individuals who maintain stable function, those who decline, and those who improve over time—distinctions that may reflect successful management of shared cardiometabolic risk factors [4,[13], [14], [15]]. Conversely, declining trajectories may signal progressing systemic pathology. Currently, the prognostic value of these distinct longitudinal patterns for all-cause mortality in a dedicated CVD cohort remains insufficiently characterized.

Addressing this gap requires robust methodological consideration, as defining trajectories from limited data poses challenges in classification stability. Furthermore, a global cognitive score may mask divergent trends across specific domains. Therefore, a comprehensive approach must combine rigorous trajectory modeling with domain-specific examination.

Leveraging data from the China Health and Retirement Longitudinal Study (CHARLS), this prospective study aims to identify distinct multi-year cognitive trajectories in middle-aged and older adults with CVD using a data-driven clustering framework, and to evaluate their independent association with all-cause mortality. The mean follow-up period was 4.7 years.

## 2 Methods

### 2.1 Study population

This study utilized data from the China Health and Retirement Longitudinal Study (CHARLS), a prospective, nationally representative study of adults aged 45 and older in China. The baseline national survey (Wave 1) was implemented in 2011, recruiting 17,708 middle-aged and older adults residing in 450 villages located within 150 districts across 28 Chinese provinces. Standardized questionnaires were employed to gather demographic and clinical data, and subsequent follow-up waves were executed every two to three years to capture evolving health trajectories. We utilized five CHARLS waves (2011, 2013, 2015, 2018, and 2020); comprehensive methodological details have been published elsewhere [16]. The study protocol received ethical clearance from the Peking University Biomedical Ethics Review Committee (IRB00001052-11015), and written informed consent was obtained from all participants, and all procedures were conducted in accordance with the ethical standards of the 1975 Declaration of Helsinki.

**Fig. 1:**
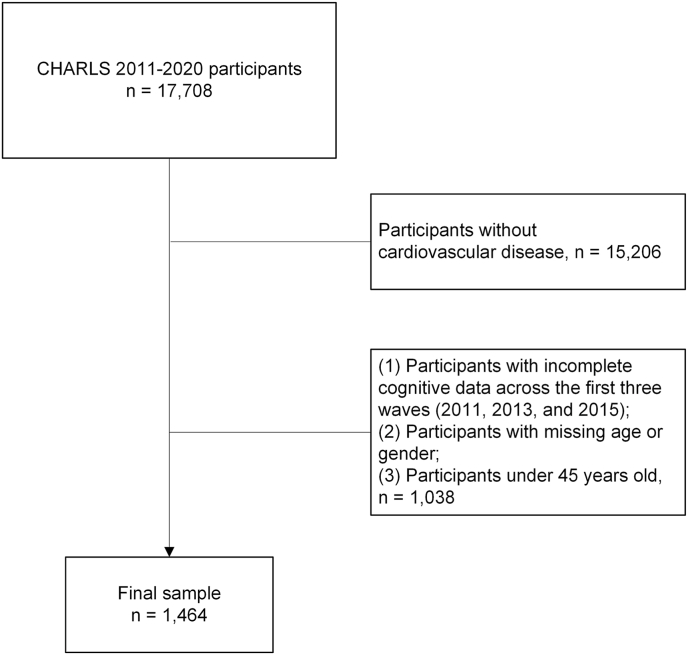
Flow chart of the study. CHARLS, the China Health and Retirement Longitudinal Study.

Participants with incomplete cognitive data across the first three waves were excluded. Participants aged <45 years were excluded. Participants without history of heart disease or stroke were excluded. Middle-aged and older adults with CVD were defined as those who (1) reported a prior diagnosis of heart disease or stroke and (2) were aged ≥45 years. After exclusions, 1464 participants were included (Fig. 1).

### 2.2 Covariates

Covariates included sociodemographic characteristics (age, sex, smoking status, alcohol consumption), health indicators (systolic blood pressure [SBP], diastolic blood pressure [DBP], and self-reported history of hypertension, diabetes, and dyslipidemia), laboratory biomarkers (fasting blood glucose [FBG], high-density lipoprotein cholesterol [HDL-C], low-density lipoprotein cholesterol [LDL-C], and glycated hemoglobin [HbA1c]), and frailty index. The numbers and percentages of participants with missing values were in Table S1.

Hypertension was defined as SBP ≥140 mmHg, DBP ≥90 mmHg, or a self-reported physician diagnosis. Diabetes was defined as FBG ≥7.0 mmol/L, HbA1c ≥ 6.5 %, or self-reported diagnosis. Dyslipidemia was defined as total cholesterol ≥240 mg/dL, triglycerides ≥150 mg/dL, LDL-C ≥ 160 mg/dL, or a self-reported history of the condition. Frailty index was defined using the Rockwood frailty index based on cumulative deficit model, calculated as the ratio of present deficits to total measured items (32-item version), with higher values indicating greater frailty severity [[17], [18], [19]].

### 2.3 Cognitive function assessment

Cognitive function was assessed at three time points: 2011, 2013, and 2015. CHARLS operationalized global cognition through four theoretically distinct domains: episodic memory, temporal orientation, attention/executive control, and visuospatial processing.

Episodic memory was measured with a ten-word learning paradigm. An interviewer read aloud a list of ten common nouns; participants immediately restated as many as they could (immediate recall). After a 4-min distractor interval, they attempted to retrieve the same items again (delayed recall). Consistent with prior recommendations, the episodic-memory score was calculated as the mean number of correctly recalled words across immediate and delayed trials, yielding a 0–10 scale.

Temporal orientation and attention were assessed with a ten-item adaptation of the Telephone Interview for Cognitive Status (TICS). Items comprised the accurate verbal report of the current date (month, day, year), day of the week, and season, as well as serial subtractions of seven from 100 up to five steps. Each correct response received one point, producing a TICS sub-score from 0 to 10.

Visuospatial constructional ability was evaluated through the intersecting-pentagons copying task. Participants were shown a stimulus consisting of two overlapping pentagons and instructed to reproduce it. Successful completion was scored 1; failure received 0.

Following standard practice in CHARLS publications [[20], [21], [22]], the three sub-domain scores were summed to yield a global cognitive-status index (range 0–21; Cronbach's α = 0.835), with higher values indicating better cognitive functioning.

### 2.4 Identification of cognitive function trajectories

To identify distinct longitudinal patterns of cognitive function, we employed a Longitudinal K-means clustering algorithm. Unlike traditional model-based approaches (e.g., Latent Growth Mixture Modeling) that assume specific functional forms (linear or quadratic) and distributional properties, this non-parametric, distance-based method offers greater flexibility in detecting irregular trajectory shapes without imposing rigid structural assumptions.

Input Data: The clustering was applied to a longitudinal data matrix of dimensions N×3, where each row represents a participant's vector of global cognitive scores across the three waves. This structure preserves the temporal dependencies inherent in the data.

Distance Metric: To explicitly handle the longitudinal nature of the data, we utilized Dynamic Time Warping (DTW) as the distance metric instead of standard Euclidean distance. DTW aligns temporal sequences to measure the similarity between trajectory shapes, ensuring that individuals are grouped based on the evolution of their cognitive function over time rather than static point-wise differences.

Model Selection: To ensure robustness, we compared this approach against standard K-means and Spectral clustering. The optimal number of clusters was determined using a two-stage evaluation: (1) Statistical Validation: We calculated standard internal validity indices (Silhouette Coefficient, Calinski-Harabasz Index, Davies-Bouldin Index) and a specialized Trajectory Silhouette Coefficient (longSil). The longSil metric specifically utilizes time-series distances to evaluate how well a trajectory fits within its assigned cluster relative to others. (2) Clinical Interpretability: The final model selection was guided by the consensus of these metrics alongside the clinical meaningfulness of the identified patterns in the context of survival and trend analyses.

### 2.5 Statistical Validation of trajectories

To quantitatively validate and characterize the identified cognitive trajectories, linear mixed-effects models (LMM) were employed. For each clustering solution (K = 2, 3, 4), separate LMMs were fitted for individual trajectory groups with total cognition score as the dependent variable. The models included time (in years) as the primary fixed effect to estimate the annual rate of cognitive change. For each trajectory group, we tested the null hypothesis that the annual slope equals zero ($H_{0}:slope=0$) against the alternative hypothesis of non-zero change ($H_{1}:slope\neq 0$).

Based on absolute statistical characteristics, trajectory groups were assigned descriptive labels using a two-dimensional classification system including baseline cognitive level classification and cognitive change pattern classification.

The annual rates of cognitive change across subdomains by identified trajectory group were also estimated based on linear mixed-effects models.

### 2.6 Outcome ascertainment

The primary outcome was all-cause mortality. Vital status information was collected through exit interviews conducted in the 2018 and 2020 waves. The follow-up time for each participant was calculated from the date of the baseline interview in 2011 to the date of death.

### 2.7 Statistical analysis

Baseline characteristics of the study population were presented according to mortality status and cognitive trajectory group. Continuous variables were expressed as means and standard deviations (SD) and compared using t-tests or analysis of variance (ANOVA). Categorical variables were expressed as numbers and percentages (%) and compared using the chi-squared test.

To avoid immortal time bias introduced by the period required to define cognitive trajectories (2011–2015), we employed a Landmark Analysis method. The landmark point was set at Wave 3 (2015). Consequently, the follow-up period for survival analysis commenced from the interview date in 2015 and continued until the date of death or the censoring date. Cox proportional hazards models were used to estimate hazard ratios (HRs) and 95 % confidence intervals (CIs). Schoenfeld residuals were used to test the proportional hazards assumption, and no violation was observed. Covariates with concerning collinearity observed were excluded. Three hierarchical models were constructed: Model 1 (Crude), Model 2 (Partially Adjusted for age and sex), and Model 3 (Fully Adjusted for age, sex, BMI, drinking status, smoking status, hypertension, dyslipidemia, and diabetes). In the event of missing values, the multiple imputation method was employed to impute the variables. We assumed data were missing at random (MAR). 5 imputed datasets were generated. The imputation model included all covariates used in the analysis, as well as the outcome variable (mortality status) and the cumulative hazard to preserve the relationship between covariates and survival time. The Kaplan-Meier (KM) curve was used to depict the relationship between cognitive function trajectories and all-cause mortality, and group differences were tested using the log-rank test.

Further subgroup analyses were conducted according to age, gender, smoking status, BMI, drinking status, smoking status, hypertension, dyslipidemia, and diabetes. A likelihood ratio test was conducted to assess the interaction between subgroups and cognitive function trajectories.

Various sensitivity analyses were also conducted. (1) To mitigate the potential impact of reverse causation, participants who died in the first two years of their follow-up were excluded. (2) Analyses were repeated only for participants with complete data. (3) To investigate a potential role of white blood cell count, mean corpuscular volume (MCV), blood urea nitrogen (BUN), glucose, creatinine, HDL-cholesterol, LDL-cholesterol, c-reactive protein (CRP), and uric acid with any of the observed associations, we further adjusted for these laboratory indicators. (4) To investigate a potential role of end-stage health decline, we further adjusted for frailty index. (5) To assess potential selection bias from excluding patients with incomplete cognitive data, we compared baseline characteristics between complete and incomplete cognitive data groups.

All statistical analyses were performed using Python (version 3.9) and R (version 4.3). A two-sided P-value <0.05 was considered statistically significant.

## 3 Results

### 3.1 Selection of the optimal clustering solution

A consensus of these metrics indicated that clustering quality generally decreased as the number of clusters increased beyond an optimal solution at K = 2 (Fig. S1, and Table S2). However, to balance statistical robustness with clinical interpretability, we selected the K = 4 solution for our primary analysis as it offered a more granular and clinically nuanced stratification of cognitive decline. To ensure the robustness of our findings, all main analyses were replicated using the K = 2 and K = 3 solutions, the results of which were provided in the supplementary materials.

Across the evaluated solutions for K = 2, 3, and 4, the Longitudinal K-means (DTW) algorithm consistently demonstrated superior or highly competitive performance across a majority of these metrics. Notably, for the K = 4 solution, which was prioritized for its potential clinical interpretability, Longitudinal K-means (DTW) yielded the most favorable scores on the Calinski-Harabasz Index and the longSil metric (Fig. S1, and Table S2). Therefore, to ensure methodological consistency across our primary and sensitivity analyses, the Longitudinal K-means (DTW) algorithm was selected as the optimal method for classifying cognitive trajectories for all subsequent analyses.

### 3.2 Characterization of cognitive trajectories

**Fig. 2:**
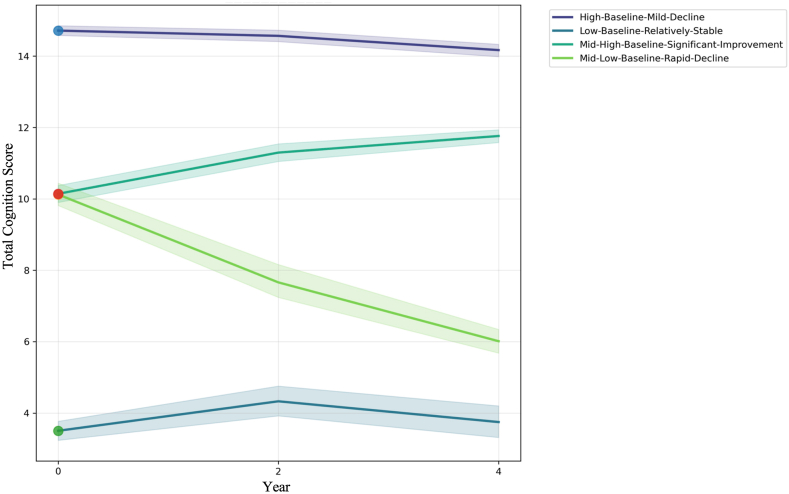
Mean cognitive trajectories identified by K-means clustering among middle-aged and older Chinese adults with cardiovascular disease in CHARLS (2011–2015). The plot illustrates four distinct cognitive trajectory groups derived from K-means clustering of total cognition scores across three waves. Each line represents the mean total cognition score for a specific trajectory group, with the shaded areas indicating the 95 % confidence intervals.

LMM validated the distinct nature of trajectories identified by the Longitudinal K-means (DTW) algorithm. For our primary K = 4 solution, four distinct trajectories were characterized based on their unique combination of baseline cognitive level and annual rate of change (Fig. 2, and Table 1):

**Table 1:** Estimated baseline cognitive levels and annual rates of change in global cognition across identified trajectory groups.

Metric	Low-Baseline-Relatively-Stable	P-value	Mid-Baseline-Rapid-Decline	P-value	High-Baseline-Relatively-Stable	P-value	Mid-High-Baseline-Significant-Improvement	P-value
Baseline level (Intercept)	3.737 (0.150)	<0.001	9.987 (0.210)	<0.001	14.756 (0.080)	<0.001	10.257 (0.126)	<0.001
Annual rate of change (Slope)	0.061 (0.069)	0.376	−1.028 (0.083)	<0.001	−0.137 (0.025)	<0.001	0.405 (0.049)	<0.001

**High-Baseline-Relatively-Stable (N** = **573):** This group began with the highest cognitive function (mean baseline: 14.76 points) and exhibited a mild but statistically significant decline (Slope: −0.14 points/year, p < 0.001). The trajectory represents the largest group (39.1 % of cohort) and demonstrates slightly gradual cognitive deterioration from an elevated baseline.

**Mid-High-Baseline-Significant-Improvement (N** = **457):** This group started with a high-moderate cognitive function (mean baseline: 10.26 points) and showed significant cognitive enhancement (Slope: +0.40 points/year, p < 0.001). 31.2 % of cohort was in this positive trajectory.

**Mid-Baseline-Rapid-Decline (N** = **244):** This group experienced the most severe cognitive deterioration (Slope: −1.03 points/year, p < 0.001) from a moderate baseline level (mean: 9.99 points). 16.7 % of the cohort was in this trajectory.

**Low-Baseline-Relatively-Stable (N** = **190):** This group started with the lowest cognitive function (mean baseline: 3.74 points) and remained relatively stable over the observation period (Slope: +0.06 points/year, p = 0.38). 13.0 % of the cohort was in this trajectory.

These trajectory patterns were consistent across alternative clustering solutions. The K = 3 solution identified High-Baseline-Relatively-Stable (N = 707), Mid-Baseline-Rapid-Decline (N = 493), and Low-Baseline-Moderate-Decline (N = 264) trajectories, while the K = 2 solution distinguished between High-Baseline-Relatively-Stable (N = 1001) and Low-Baseline-Mild-Decline (N = 463) groups (Fig. S2, Tables S3 and S4). Across all solutions, trajectories with significant cognitive decline were consistently identified through absolute testing (all p < 0.05).

### 3.3 Cognitive subdomain contributions to trajectory patterns

To elucidate the specific cognitive domains driving the observed trajectory patterns, we conducted absolute significance testing for three key subdomains by LMM: Visuospatial processing, Temporal orientation and attention, and Episodic memory within each trajectory group (Table 2).

**Table 2:** Estimated annual rates of cognitive change across subdomains by identified trajectory group based on linear mixed-effects models.

Cognitive Subdomain	Low-Baseline-Relatively-Stable	P-value	Mid-Baseline-Rapid-Decline	P-value	High-Baseline-Relatively-Stable	P-value	Mid-High-Baseline-Significant-Improvement	P-value
Visuospatial processing	−0.008 (0.010)	0.386	−0.047 (0.010)	<0.001	−0.012 (0.005)	0.014	−0.008 (0.007)	0.279
Temporal orientation and attention	0.030 (0.041)	0.461	−0.573 (0.049)	<0.001	−0.085 (0.016)	<0.001	0.215 (0.028)	<0.001
Episodic memory	−0.115 (0.032)	<0.001	−0.291 (0.031)	<0.001	−0.044 (0.019)	0.018	0.075 (0.021)	<0.001

For the High-Baseline-Relatively-Stable group, Temporal orientation and attention showed the most significant deterioration (Slope: −0.081 points/year, p < 0.001), followed by moderate decline in Episodic memory (Slope: −0.042 points/year), while Visuospatial processing remained relatively stable (Slope: −0.012 points/year, p = 0.008).

The Mid-Baseline-Rapid-Decline group exhibited the most severe and widespread deterioration across all domains. Temporal orientation and attention demonstrated the steepest decline (Slope: −0.401 points/year, p < 0.001), representing the primary driver of rapid cognitive deterioration. Episodic memory showed substantial decline (Slope: −0.307 points/year, p < 0.001) as a secondary contributor, while Visuospatial processing showed moderate deterioration (Slope: −0.055 points/year, p < 0.001).

The Mid-High-Baseline-Significant-Improvement group demonstrated selective enhancement across domains. Temporal orientation and attention showed the most significant improvement (Slope: +0.176 points/year, p < 0.001). Episodic memory exhibited moderate improvement (Slope: +0.073 points/year, p < 0.001), while visuospatial processing remained relatively stable (Slope: −0.014 points/year).

The Low-Baseline-Relatively-Stable group showed heterogeneous patterns across subdomains. Remarkably, Temporal orientation and attention demonstrated significant improvement (Slope: +0.307 points/year). However, Episodic memory showed moderate decline (Slope: −0.086 points/year), while Visuospatial processing remained stable (Slope: −0.000 points/year).

### 3.4 Cognitive trajectories and baseline characteristics

The analysis included 1464 participants with baseline CVD. The mean age was 60.3 (SD 9.1) years, and 41.9 % were male.

Baseline characteristics of the study population by cognitive trajectory are presented in Table 3; baseline characteristics by mortality status are provided in Table S5.

**Table 3:** Baseline characteristics of middle-aged and older Chinese adults with cardiovascular disease by cognitive trajectory in CHARLS 2011–2020.

Characteristic	Overall (N = 1464)	High-Baseline-Relatively-Stable	Low-Baseline-Relatively-Stable	Mid-Baseline-Rapid-Decline	Mid-High-Baseline-Significant-Improvement	P-Value
Age, mean (SD)	60.3 (9.1)	59.0 (8.6)	63.3 (9.6)	62.1 (9.5)	59.8 (8.9)	<0.001
Sex, n (%)						<0.001
Male	614 (41.9)	292 (51.0)	25 (13.2)	87 (35.7)	210 (46.0)	
Current Drinker, n (%)	362 (24.7)	176 (30.7)	15 (7.9)	51 (20.9)	120 (26.3)	<0.001
Current Smoker, n (%)	551 (37.6)	233 (40.7)	41 (21.6)	83 (34.0)	194 (42.5)	<0.001
Hypertension, n (%)	744 (50.8)	287 (50.1)	100 (52.6)	121 (49.6)	236 (51.6)	0.879
Dyslipidemia, n (%)	351 (24.0)	165 (28.8)	42 (22.1)	51 (20.9)	93 (20.4)	0.011
Diabetes, n (%)	184 (12.6)	86 (15.0)	21 (11.1)	33 (13.5)	44 (9.6)	0.074
BMI, mean (SD)	24.6 (4.3)	25.1 (4.2)	23.7 (4.4)	24.1 (3.9)	24.8 (4.4)	<0.001

Abbreviations: CVD, cardiovascular disease; CHARLS, the China Health and Retirement Longitudinal Study.

Significant differences were observed across cognitive trajectory groups for the majority of demographic and health-related variables. The mean age differed significantly among groups (P < 0.001); individuals in the Low-Baseline-Relatively-Stable (63.3 years) and Mid-Baseline-Rapid-Decline (62.1 years) trajectories tended to be older compared to those in the High-Baseline-Relatively-Stable (59.0 years) and Mid-High-Baseline-Significant-Improvement (59.8 years) groups. The proportion of males varied significantly across trajectories (P < 0.001), with the High-Baseline-Relatively-Stable group having the highest proportion of males (51.0 %) and the Low-Baseline-Relatively-Stable group having the lowest (13.2 %). The prevalence of current drinking and smoking also showed significant variation (both P < 0.001), with the High-Baseline-Relatively-Stable group exhibiting the highest prevalence of current drinking (30.7%) and the Mid-High-Baseline-Significant-Improvement group showing the highest proportion of current smokers (42.5%). Significant differences were also noted in BMI (P < 0.001), with the High-Baseline-Relatively-Stable group having the highest mean BMI (25.1 kg/m²). Regarding chronic conditions, the prevalence of dyslipidemia varied significantly (P = 0.011), being highest in the High-Baseline-Relatively-Stable group (28.8%). However, no significant differences were observed across the trajectory groups regarding the prevalence of hypertension (P = 0.879) or diabetes (P = 0.074).

Mid-High-Baseline-Significant-Improvement.

High-Baseline-Relatively-Stable trajectory showing the highest rates of current drinkers (30.7 %). While hypertension prevalence was similar across groups (P = 0.879), dyslipidemia differed significantly (P = 0.011), with the High-Baseline-Relatively-Stable group having the highest prevalence (28.8 %). Additionally, mean BMI differed significantly among groups (P < 0.001), with the High-Baseline-Relatively-Stable trajectory having the highest mean BMI (25.1).

### 3.5 Association between cognitive trajectories and mortality

As shown in Table 4, distinct cognitive trajectories demonstrated differential associations with all-cause mortality risk when compared to the Mid-High-Baseline-Significant-Improvement trajectory, which served as the reference group.

**Table 4:** Multivariable cox regression models examining the association of cognitive trajectory with all-cause mortality among middle-aged and older Chinese adults with cardiovascular disease in CHARLS 2011–2020.

Cognitive Trajectory	HR (95 % CI)					
	Model 1	P-value	Model 2	P-value	Model 3	P-value
High-Baseline-Relatively-Stable	0.75 (0.49–1.15)	0.187	0.75 (0.49–1.15)	0.193	0.74 (0.48–1.13)	0.163
Mid-High-Baseline-Significant-Improvement	1 [Reference]		1 [Reference]		1 [Reference]	
Mid-Baseline-Rapid-Decline	2.07 (1.37–3.13)	<0.001	1.97 (1.29–2.99)	0.002	2.01 (1.32–3.05)	0.001
Low-Baseline-Relatively-Stable	1.62 (1.02–2.60)	0.043	1.81 (1.10–2.98)	0.02	1.82 (1.10–3.00)	0.019

Abbreviations: CHARLS, the China Health and Retirement Longitudinal Study.

Model 1 was adjusted for none.

Model 2 was adjusted for age, sex.

Model 3 was further adjusted for age, sex, BMI, drinking status, smoking status, hypertension, dyslipidemia, and diabetes.

In the crude model (Model 1), both the Mid-Baseline-Rapid-Decline trajectory (HR = 2.07, 95 % CI: 1.37–3.13; P < 0.001) and the Low-Baseline-Relatively-Stable trajectory (HR = 1.62, 95 % CI: 1.02–2.60; P = 0.043) were associated with significantly higher risks of mortality. Conversely, the High-Baseline-Relatively-Stable trajectory showed a non-significant protective association (HR = 0.75, 95 % CI: 0.49–1.15; P = 0.187).

These associations persisted after adjusting for age and sex (Model 2) and in the fully adjusted model (Model 3), which additionally controlled for BMI, drinking status, smoking status, hypertension, dyslipidemia, and diabetes. In the fully adjusted model, the hazard ratios for mortality remained significantly elevated for both the Mid-Baseline-Rapid-Decline group (HR = 2.01, 95 % CI: 1.32–3.05; P = 0.001) and the Low-Baseline-Relatively-Stable group (HR = 1.82, 95 % CI: 1.10–3.00; P = 0.019). The High-Baseline-Relatively-Stable group continued to show no significant association with mortality in any model (fully adjusted HR = 0.74, 95 % CI: 0.48–1.13; P = 0.163).

**Fig. 3:**
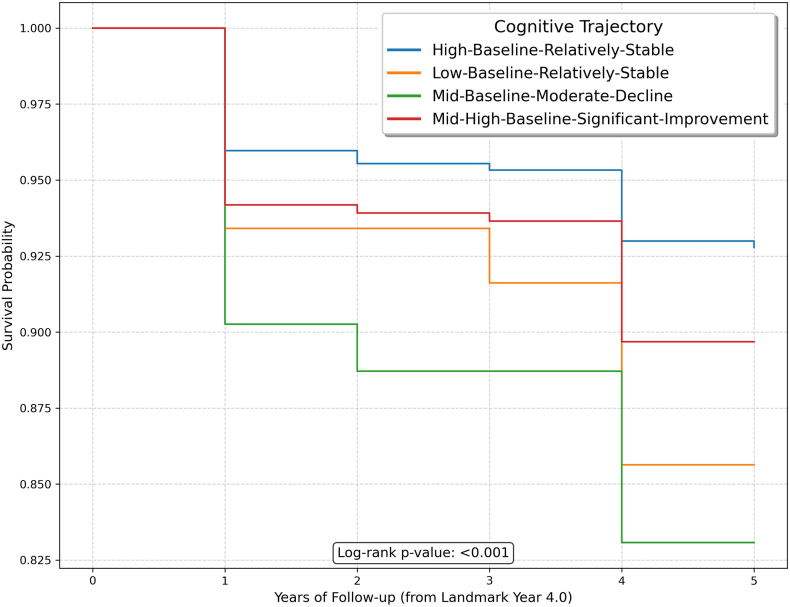
Kaplan-Meier survival curves for all-cause mortality stratified by cognitive trajectory among middle-aged and older Chinese adults with cardiovascular disease in CHARLS (2011–2020). A log-rank test indicated significant differences in survival probabilities among the groups, p < 0.001.

KM survival analysis also corroborated these results (Fig. 3). Further analyses using K = 2 and K = 3 clustering solutions were consistent with the results from K = 4 (Fig. S3, Tables S6 and S7).

### 3.6 Subgroup analyses

Subgroup analyses were conducted to evaluate the consistency of the association between cognitive trajectories and all-cause mortality across key demographic and clinical strata (Table 5). Statistically significant interactions were identified for hypertension status (P for interaction = 0.009) and BMI category (P for interaction = 0.044). The association between unfavorable cognitive trajectories and mortality was substantially stronger among participants with hypertension. Specifically, in the hypertensive group, the hazard ratios for the Mid-Baseline-Rapid-Decline and Low-Baseline-Relatively-Stable trajectories were 3.46 (95 % CI: 1.98–6.03) and 2.55 (95 % CI: 1.30–4.98), respectively, relative to the reference group. In contrast, these associations were attenuated and not statistically significant among participants without hypertension.

**Table 5:** Stratified analyses of the associations between cognitive trajectory and all-cause mortality among middle-aged and older Chinese adults with cardiovascular disease in CHARLS 2011–2020.

Characteristic	N (Events)	HR (95 % CI)				P for
		High-Baseline-Relatively-Stable	Mid-High-Baseline-Significant-Improvement	Mid-Baseline-Rapid-Decline	Low-Baseline-Relatively-Stable	Interaction
Age						0.887
<65 years	1001 (65)	0.77 (0.41–1.44)	1 [Reference]	1.97 (0.99–3.90)	1.87 (0.84–4.14)	
≥65 years	463 (96)	0.67 (0.37–1.22)	1 [Reference]	2.00 (1.16–3.44)	1.85 (0.96–3.53)	
Sex						0.97
Male	614 (100)	0.79 (0.48–1.30)	1 [Reference]	1.87 (1.11–3.16)	1.61 (0.73–3.58)	
Female	850 (61)	0.64 (0.27–1.51)	1 [Reference]	2.06 (1.00–4.25)	2.18 (1.06–4.47)	
Current Drinker						0.846
Yes	362 (45)	0.63 (0.29–1.37)	1 [Reference]	1.86 (0.85–4.08)	2.50 (0.73–8.58)	
No	1102 (116)	0.76 (0.45–1.28)	1 [Reference]	1.96 (1.19–3.23)	1.69 (0.97–2.94)	
Current Smoker						0.69
Yes	551 (85)	0.64 (0.37–1.13)	1 [Reference]	1.49 (0.85–2.63)	1.57 (0.76–3.26)	
No	913 (76)	0.95 (0.48–1.88)	1 [Reference]	2.89 (1.49–5.58)	2.35 (1.13–4.88)	
BMI Category						0.044
<24 kg/m^2^	633 (86)	0.71 (0.39–1.29)	1 [Reference]	1.76 (0.97–3.17)	1.89 (0.97–3.69)	
24–28 kg/m^2^	592 (53)	1.27 (0.53–3.08)	1 [Reference]	5.08 (2.19–11.80)	3.81 (1.38–10.52)	
>28 kg/m^2^	239 (22)	0.45 (0.17–1.20)	1 [Reference]	0.75 (0.19–3.05)	0.28 (0.03–2.33)	
Hypertension						0.009
Yes	745 (95)	0.81 (0.44–1.48)	1 [Reference]	3.46 (1.98–6.03)	2.55 (1.30–4.98)	
No	719 (66)	0.64 (0.35–1.17)	1 [Reference]	0.80 (0.40–1.63)	1.17 (0.53–2.56)	
Diabetes						0.161
Yes	184 (33)	0.32 (0.12–0.89)	1 [Reference]	2.14 (0.85–5.41)	1.07 (0.30–3.82)	
No	1280 (128)	0.90 (0.56–1.44)	1 [Reference]	1.90 (1.18–3.06)	2.07 (1.18–3.63)	
Dyslipidemia						0.084
Yes	352 (34)	0.35 (0.13–0.91)	1 [Reference]	2.14 (0.91–5.04)	0.99 (0.30–3.27)	
No	1112 (127)	0.92 (0.57–1.50)	1 [Reference]	2.02 (1.24–3.29)	2.11 (1.20–3.68)	

Abbreviations: CVD, cardiovascular disease; CHARLS, the China Health and Retirement Longitudinal Study.

Adjusted for age, sex, BMI, drinking status, smoking status, hypertension, dyslipidemia, and diabetes. The strata variable was not included in the model when stratifying by itself.

The interaction with BMI category revealed that the mortality risk associated with cognitive decline was most pronounced in the overweight population. Participants with a BMI of 24–28 kg/m^2^ exhibited the highest risk in the Mid-Baseline-Rapid-Decline group (HR: 5.08, 95 % CI: 2.19–11.80) compared to those in other BMI categories. Regarding other covariates, the associations were largely consistent across strata of age, sex, smoking status, and alcohol consumption, with no significant interactions observed (P for interaction >0.05). Similarly, while diabetes and dyslipidemia are common comorbidities, formal interaction tests for these conditions did not reach statistical significance (P = 0.161 and P = 0.084, respectively).

### 3.7 Sensitivity analyses

The results of sensitivity analyses supported the robustness of the primary findings. In the sensitivity analysis restricted to complete cases, the association between the Low-Baseline-Relatively-Stable and the Mid-Baseline-Rapid-Decline trajectories and all-cause mortality remained significantly associated with mortality (Table S8). After excluding participants who died within the first two years of follow-up to account for potential reverse causality, the associations remained strong and statistically significant (Table S9). After further adjusted by laboratory parameters including white blood cell count, mean corpuscular volume (MCV), blood urea nitrogen (BUN), glucose, creatinine, HDL-cholesterol, LDL-cholesterol, c-reactive protein (CRP), and uric acid, the results remained stable (Table S10). After further adjusted by frailty index to account for the potential confounding effects of end-stage health decline, the results remained stable (Table S10). Sensitivity analysis of cognitive data completeness revealed that patients with incomplete cognitive data were older and had higher glucose, CRP, hypertension, and diabetes prevalence (Table S11).

## 4 Discussion

In this prospective study of Chinese middle-aged and older adults with established CVD, we identified four distinct cognitive trajectories and found that patterns of “Mid-Baseline-Rapid-Decline” or “Low-Baseline-Relatively-Stable” cognitive function are powerful predictors of all-cause mortality, independent of a wide range of traditional risk factors. Additionally, it is noteworthy that in the “Mid-High-Baseline-Significant-Improvement” trajectory group, despite patients starting with moderate cognitive levels, no significant increase in CVD mortality risk was observed.

### 4.1 Overview of principal findings

As previously mentioned, most prior studies have focused on the relationship between baseline cognitive function and the incidence and prognosis of CVD [[7], [8], [9], [10]]. However, cognitive decline is a dynamic process in middle-aged and older adults, and assessing only baseline cognitive function may overlook important information. Our findings further highlight this point, demonstrating that patients with persistently low or rapid-declining cognitive trajectories exhibit an elevated risk of mortality, whereas no significant increase in mortality risk was observed in those with a moderate-increasing trajectory.

The “Mid-Baseline-Rapid-Decline” group, comprising 16.7 % of the cohort, represents a phenotype of what we term “malignant” cognitive aging. The annual rate of decline in this group (−1.03 points/year) is clinically profound and distinct from normative aging. To contextualize this magnitude, in large longitudinal cohorts such as the Health and Retirement Study,latent variable analyses have demonstrated that normative age-related decline is primarily driven by episodic memory, with an estimated annual loss of approximately -0.10 points on a 0-10 scale, while other mental status domains remain relatively stable [23]. Thus, the deterioration observed in this specific trajectory proceeds at a velocity roughly 10 times faster than expected age-related changes. This signifies a pathological collapse of cognitive reserve rather than normative senescence.

Conversely, with the similar initial points to the “Mid-Baseline-Rapid-Decline” group, the “Mid-High-Baseline-Significant-Improvement” group (31.2 %) exhibited an upward trajectory (+0.40 points/year). While improvement in longitudinal cognitive testing is often attributed to practice effects (the “learning curve” of repeated testing) [24], the magnitude of improvement here may suggest a component of genuine cognitive resilience or “cognitive reserve".

Clinically, the absence of elevated mortality risk in this group confirms that this trajectory represents a benign or resilient phenotype, distinguishing it from the pathological decline groups. This distinction is crucial for clinicians: stable or improving cognition in a CVD patient is a potent positive prognostic marker, signaling preserved physiological resilience.

The Low-Baseline-Relatively-Stable group (13.0 %) presents a different clinical picture. These individuals began with profoundly low scores (mean 3.74) and remained there. Demographically, this group was older, and the highest proportion of females. Their high mortality risk (HR = 1.82) mirrors that of the rapid decliners, yet the trajectory is flat.

This group likely represents individuals with established, severe structural brain disease (advanced dementia or severe post-stroke impairment) entering the study. Their mortality risk is driven by the frailty and vulnerability associated with advanced cognitive failure.

Crucially, the adjustment for the Frailty Index in our sensitivity analysis (Table S10) confirmed that the observed risk is specific to cognitive trajectory and not a result of physiological decline.

### 4.2 Potential mechanisms and modifying factors

We observed a significant interaction for hypertension (P for interaction = 0.009) and BMI category (P for interaction = 0.044). For patients with hypertension, the mortality risk of the “Mid-Baseline-Rapid-Decline” group was over three times higher (HR = 3.46) compared to normotensive controls. Hypertension causes mechanical stress that leads to arteriolosclerosis, impairing neurovascular coupling and cerebral perfusion [25,26]. For BMI, we found that the mortality risk associated with cognitive decline was most pronounced in the overweight population (BMI 24–28 kg/m^2^), with an HR of 5.08.

Mechanistically, these findings may be attributed to the fact that, although certain risk factors for cognitive decline—such as age and genetics—are non‐modifiable, cognitive function and CVD share many modifiable risk factors, such as hypertension, obesity, smoking, physical inactivity, and diabetes [4,27,28]. Therefore, controlling these risk factors may concurrently protect both cardiovascular health and cognitive function.

Previous studies have elucidated the potential mechanisms through which these shared risk factors impair cognitive function. Risk factors such as diabetes, hypertension, obesity, and smoking contribute to a chronic systemic inflammatory state, promote oxidative stress, and induce vascular endothelial dysfunction, thereby leading to cerebral microangiopathy and impaired cerebral blood flow regulation [[29], [30], [31], [32]]. Furthermore, these factors may accelerate β-amyloid deposition and hyperphosphorylation of tau protein, exacerbating neurodegeneration [33,34]. Metabolic disturbances including insulin resistance and dyslipidemia can also affect blood-brain barrier integrity and disrupt neurotransmitter balance, impairing synaptic plasticity and hippocampal function, ultimately resulting in cognitive decline [29].

Additionally, the impact of drug therapy must be noted. Compared to other classes, Angiotensin Receptor Blockers (ARBs) and Angiotensin-Converting Enzyme (ACE) inhibitors crossing the blood-brain barrier are associated with better memory retention [35]. Beta-blocker treatment could also reduce the risk of cognitive impairment in hypertensive older adults [36]. Concurrently, other medications, such as proton pump inhibitors (PPIs), have been demonstrated to contribute to cognitive impairment [37,38].

Due to data limitations, we were unable to measure the potential impact of medication. Future studies should quantify this effect, especially considering polypharmacy is ubiquitous in the CVD population.

### 4.3 Clinical implications: integrated heart-brain care

Although numerous studies have demonstrated a close relationship between cognitive function and CVD across multiple dimensions, cognitive assessment remains largely overlooked in the diagnosis and management of CVD. Due to limited research exploring cognitive impairment in patients with cardiovascular diseases, major vascular disease management guidelines currently do not recommend routine screening for cognitive or Alzheimer's disease outcomes [39,40]. Moreover, a recent study revealed that the prevalence of undiagnosed cognitive impairment in the CVD population is significantly higher than in the general population, with 29 % meeting the criteria for mild cognitive impairment—substantially exceeding the rate of 22 % observed in the general population. Thus, despite being at considerably higher risk for cognitive impairment, the CVD population remains underdiagnosed [41].

Our study provides further evidence to support integrated heart-brain health strategies and underscores the necessity of incorporating cognitive assessment in future intervention studies involving CVD populations.

A key question for clinicians is: “How much decline matters?” Our study indicates a decline of >1.0 point/year on the global score is a “red flag” event associated with doubled mortality. In the literature of normal aging, the annual rate of decline on TICS-based composites is estimated at approximately 0.1 points per year [23]. Some studies using the TICS-m scale indicate that a 0.9-point decline roughly corresponds to the effect of five years of physiological aging using the TICS-m scale indicate that a 0.9-point decline roughly corresponds to the effect of five years of physiological aging [42]. Therefore, the decline observed in our Mid-Baseline-Rapid-Decline group (−1.03 points/year) represents a catastrophic acceleration of aging.

Clinically, this magnitude of change is not subtle; it likely corresponds to noticeable deficits in Instrumental Activities of Daily Living (IADLs) such as managing medications, handling finances, or tracking appointments. Detection of a decline of this magnitude should trigger an immediate comprehensive geriatric assessment.

For clinical practice, another consideration is which tools to use for assessing cognitive function in CVD patients.

Subdomain analysis results indicate that “Temporal orientation and attention” and “Episodic memory” show the most significant decline in the rapid decline group (annual rates of cognitive change: −0.573 (0.049), and −0.291 (0.031), respectively), while “Visuospatial processing” exhibits the least decline (annual rates of cognitive change: −0.047 (0.010)). However, the total scores for different subdomains vary (Temporal orientation and attention: 10 points; Episodic memory: 10 points; Visuospatial processing: 1 point), so the decline in visuospatial processing cannot be overlooked.

Commonly used clinical cognitive function assessments include: Mini-Cog Performance [43], Montreal Cognitive Assessment (MoCA) [44], and the Mini Mental State Examination (MMSE) [45]. Among these, the Mini-Cog Performance lacks the dominant subdomain of decline, “Temporal orientation and attention.” Although it has the shortest screening time, it may not be suitable for assessment. Previous studies have demonstrated that compared to the MMSE, the MoCA exhibits higher sensitivity for mild cognitive impairment (MCI) and vascular cognitive impairment [46,47]. Therefore, using the MoCA to assess cognitive function changes in CVD patients in clinical settings may be more appropriate, despite its screening time.

Furthermore, since the CHARLS's recognition score we employed is an adaptation of the TICS score [[20], [21], [22]], many items differ from those in the aforementioned scores. Future research is warranted to validate the existence of a rapid decline group and determine the annual rate of change for diagnosing this group across other scores. Based on our findings, reassessing cognitive function in CVD patients every 1–2 years may be necessary.

Optimizing the management of specific cardiovascular pathologies could improve prognosis [48,49], this may improve these adverse cognitive trajectories, too.

### 4.4 Strengths and limitations

The study's strengths include its large, nationally representative sample, prospective design, and the use of trajectory modeling.

However, some limitations should be noted. Although the follow-up period was long, the observational design cannot establish causal relationships. Second, regarding the methodological approach, we initially attempted to model cognitive trajectories using parametric growth mixture modeling frameworks (e.g., Latent Class Growth Analysis). However, models specifying two to five latent classes failed to converge. This non-convergence likely stems from the strict parametric assumptions required by these models (e.g., specific polynomial growth shapes and distributional normality), which may not align with the heterogeneity and limited time points (three waves) of our data. Consequently, we adopted Longitudinal K-means with Dynamic Time Warping as a robust, non-parametric alternative. While this method effectively captures trajectory shapes without imposing rigid structural assumptions, we acknowledge that unlike growth mixture models, K-means does not account for measurement error within the model structure or provide probabilistic class assignments (soft clustering). Future studies with extended follow-up periods and more frequent assessment points may allow for the application of parametric models to further validate these findings. Third, since the data were obtained from questionnaires rather than clinical settings, we were unable to distinguish specific types of CVD in the CHARLS dataset. Fourth, although a wide range of potential covariates were adjusted for, residual confounding cannot be fully ruled out. Finally, our sensitivity analysis showed that participants excluded due to incomplete cognitive data were generally older and had a higher burden of comorbidities (Table S11), which may suggest a ‘healthy survivor’ effect and limit the generalizability of our findings to sicker populations.

## 5 Conclusion

In this prospective cohort of middle-aged and older Chinese adults with established CVD, we demonstrated that trajectory-based patterns of persistently low or declining cognitive function are independently predictive of all-cause mortality. Conversely, individuals whose cognition improved over time did not experience excess mortality despite similarly low baseline performance. These data emphasize the dynamic nature of cognitive change and suggest that modifiable, shared cardiometabolic risk factors may simultaneously shape both vascular and cerebral outcomes.

Utilizing tools like the MoCA, clinicians can detect this “malignant” trajectory early. Future research should focus on validating the existence of a rapid decline group, determining the annual rate of change for diagnosing this group across other scores, and whether reversing vascular risk factors can alter this trajectory.

## CRediT authorship contribution statement

**Xiaopeng Song:** Writing – original draft, Formal analysis, Conceptualization. **Yufei Wang:** Writing – original draft, Methodology, Data curation. **Hua Chen:** Writing – review & editing, Supervision, Project administration, Investigation, Conceptualization.

## Data availability statement

The data that support the findings of this study are available at the CHARLS Research Data Center to the entire research community free of charge (https://charls.pku.edu.cn/en/index.htm).

## Declaration of generative AI and AI-assisted technologies in the writing process

Authors declare that no generative AI/AI-assisted technologies were employed during the preparation of this work.

## Funding

This work was supported by Inner Mongolia 10.13039/501100000691Academy of Medical Sciences, Health Committee of Inner Mongolia Autonomous Region, China (grant number 2023GLLH0010).
