# Supplementary material for: The association between cognitive function trajectories and all-cause mortality in middle-aged and older Chinese adults with cardiovascular disease: A longitudinal study from CHARLS
Source: Int J Cardiol Cardiovasc Risk Prev. 2026 Jan 29;29:200583. doi: 10.1016/j.ijcrp.2026.200583 (PMC12907054; doi:10.1016/j.ijcrp.2026.200583)
Supplement: Multimedia component 1 [file mmc1.docx]

Table S1. The numbers and percentages of participants with missing values.

| Variable | Missing Count | Missing Percentage (%) |
| --- | --- | --- |
| Age | 1 | 0.07 |
| Sex | 0 | 0.00 |
| Current Drinker | 0 | 0.00 |
| Current Smoker | 0 | 0.00 |
| Hypertension | 4 | 0.27 |
| Dyslipidemia | 26 | 1.78 |
| Diabetes | 8 | 0.55 |
| Body mass index | 224 | 15.30 |
| Creatinine (mg/dl) | 324 | 22.13 |
| HbA1c (%) | 306 | 20.90 |
| LDL-cholesterol (mg/dl) | 319 | 21.79 |
| HDL-cholesterol (mg/dl) | 318 | 21.72 |
| C-reactive protein (mg/L) | 317 | 21.65 |

Figure S1. Comparison of clustering metrics for different clustering algorithms and number of clusters (*K*)


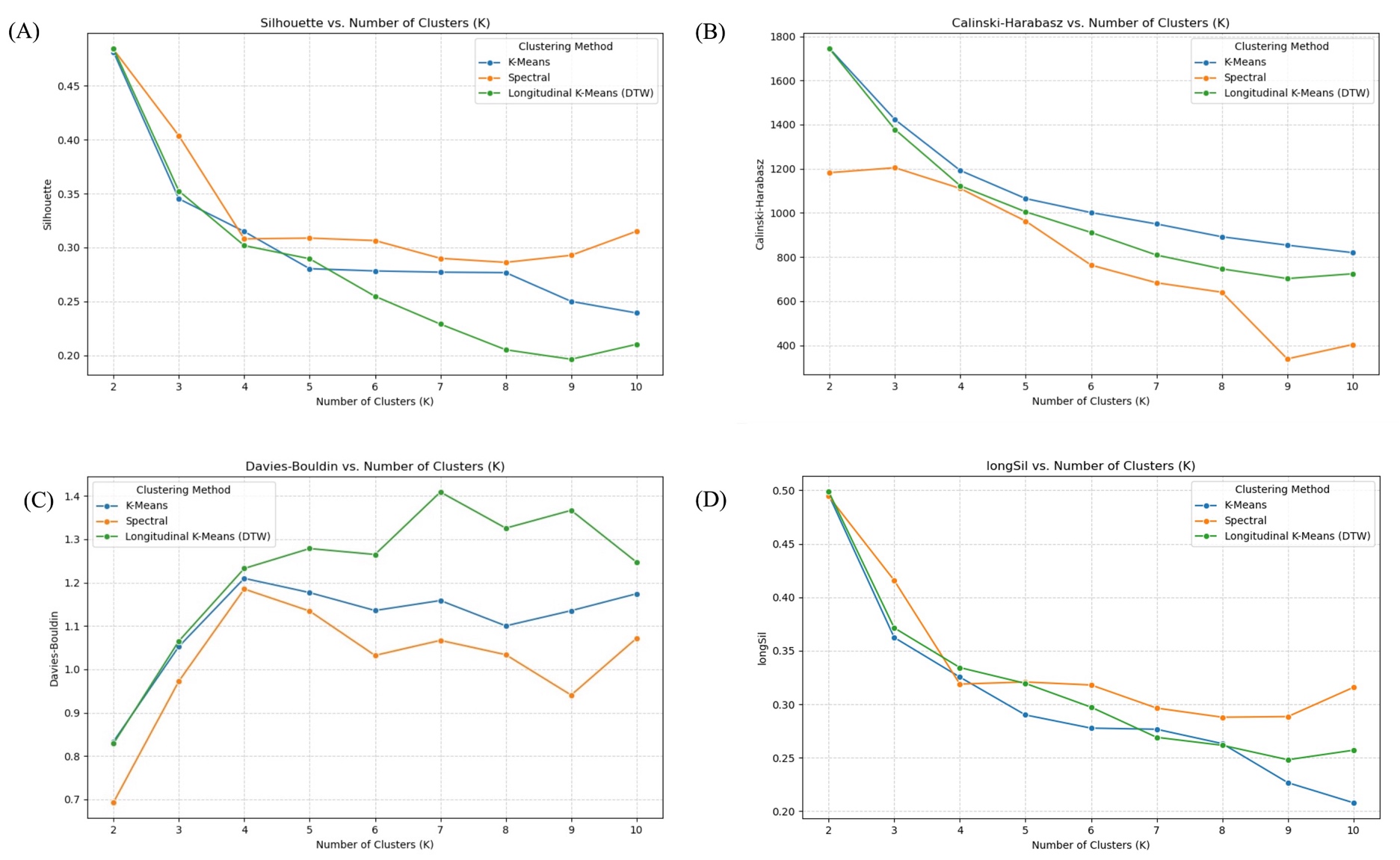


Table S2. Clustering metrics for different methods and *K* values

| Method | *K* | Silhouette | Calinski-Harabasz | Davies-Bouldin | longSil |
| --- | --- | --- | --- | --- | --- |
| K-Means | 2 | 0.4814 | 1744.8038 | 0.8335 | 0.496 |
| Spectral | 2 | 0.4837 | 1182.1066 | 0.6931 | 0.4944 |
| Longitudinal K-Means (DTW) | 2 | 0.4845 | 1743.1564 | 0.8287 | 0.4989 |
| K-Means | 3 | 0.3452 | 1422.8153 | 1.0526 | 0.3624 |
| Spectral | 3 | 0.4036 | 1204.5492 | 0.9728 | 0.4158 |
| Longitudinal K-Means (DTW) | 3 | 0.3524 | 1377.874 | 1.0652 | 0.3714 |
| K-Means | 4 | 0.3147 | 1191.9502 | 1.2097 | 0.3254 |
| Spectral | 4 | 0.3079 | 1110.4724 | 1.1852 | 0.3189 |
| Longitudinal K-Means (DTW) | 4 | 0.3017 | 1122.695 | 1.2326 | 0.3343 |
| K-Means | 5 | 0.2803 | 1064.8355 | 1.1769 | 0.29 |
| Spectral | 5 | 0.3087 | 962.4651 | 1.1344 | 0.3208 |
| Longitudinal K-Means (DTW) | 5 | 0.2895 | 1004.6841 | 1.2785 | 0.3195 |


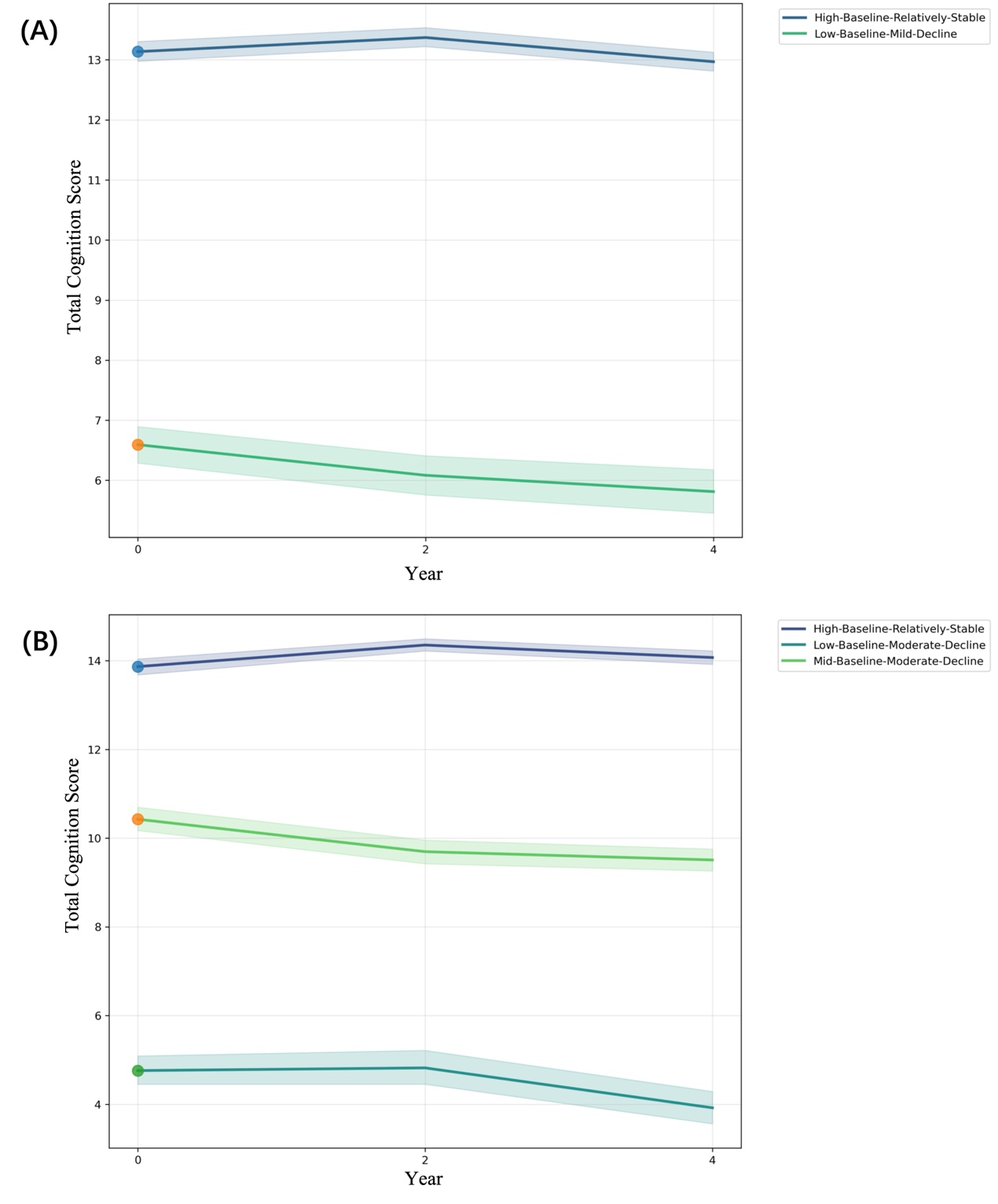


Figure S2. Mean cognitive trajectories identified by K-means clustering among middle-aged and older Chinese adults with cardiovascular disease in CHARLS (2011-2015). (A) *K*=2; (B) *K*=3*.*The plot illustrates distinct cognitive trajectory groups derived from K-means clustering of total cognition scores across three waves. Each line represents the mean total cognition score for a specific trajectory group, with the shaded areas indicating the 95% confidence intervals.

Table S3. Estimated baseline cognitive levels and annual rates of change in global cognition across trajectory groups by *K*=3.

| Metric | Low-Baseline-Moderate-Decline | P-value | Mid-Baseline-Rapid-Decline | P-value | High-Baseline-Relatively-Stable | P-value |
| --- | --- | --- | --- | --- | --- | --- |
| Baseline level (Intercept) | 4.920 (0.181) | <0.001 | 10.335 (0.126) | <0.001 | 13.996 (0.082) | <0.001 |
| Annual rate of change (Slope) | -0.210 (0.084) | 0.013 | -0.229 (0.071) | 0.001 | 0.051 (0.036) | 0.158 |

Table S4. Estimated baseline cognitive levels and annual rates of change in global cognition across trajectory groups by *K*=2.

| Metric | Low-Baseline-Mild-Decline | P-value | High-Baseline-Relatively-Stable | P-value |
| --- | --- | --- | --- | --- |
| Baseline level (Intercept) | 6.553 (0.163) | <0.001 | 13.242 (0.082) | <0.001 |
| Annual rate of change (Slope) | -0.195 (0.069) | 0.005 | -0.042 (0.032) | 0.183 |

Table S5. Baseline characteristics of middle-aged and older Chinese adults with cardiovascular disease by vital status in CHARLS 2011-2020.

| Variables | Total (n = 1,464) | Survivors (n = 1,303) | Deaths (n = 161) | *P*-value |
| --- | --- | --- | --- | --- |
| Age, mean (SD) | 60.3 (9.1) | 59.6 (8.8) | 66.3 (9.6) | <0.001 |
| Sex, n (%) |  |  |  |  |
| Female | 850 (58.1) | 789 (60.6) | 61 (37.9) | <0.001 |
| Male | 614 (41.9) | 514 (39.4) | 100 (62.1) |  |
| Current Drinker, n (%) | 362 (24.7) | 317 (24.3) | 45 (28.0) | 0.364 |
| Current Smoker, n (%) | 362 (24.7) | 304 (23.3) | 58 (36.0) | <0.001 |
| Hypertension, n (%) | 745 (50.9) | 650 (49.9) | 95 (59.0) | 0.036 |
| Dyslipidemia, n (%) | 352 (24.0) | 318 (24.4) | 34 (21.1) | 0.41 |
| Diabetes, n (%) | 184 (12.6) | 151 (11.6) | 33 (20.5) | 0.002 |
| BMI, mean (SD) | 24.7 (4.0) | 24.7 (4.0) | 23.9 (3.7) | 0.008 |

Abbreviations: CHARLS, the China Health and Retirement Longitudinal Study; BMI, body mass index.

Table S6. Multivariable cox regression models examining the association of cognitive trajectory by *K*=2 with all-cause mortality among middle-aged and older Chinese adults with cardiovascular disease in CHARLS 2011-2020.

|  | HR (95% CI) | |  |  |  |  |
| --- | --- | --- | --- | --- | --- | --- |
| Cognitive Trajectory | Model 1 | P-value | Model 2 | P-value | Model 3 | P-value |
| High-Baseline-Relatively-Stable | 1 [Reference] | | 1 [Reference] | | 1 [Reference] | |
| Low-Baseline-Relatively-Stable | 2.06 (1.51-2.81) | <0.001 | 2.06 (1.48-2.86) | <0.001 | 2.08 (1.49-2.90) | <0.001 |

Table S7. Multivariable cox regression models examining the association of cognitive trajectory by *K*=3 with all-cause mortality among middle-aged and older Chinese adults with cardiovascular disease in CHARLS 2011-2020.

|  | HR (95% CI) | |  |  |  |  |
| --- | --- | --- | --- | --- | --- | --- |
| Cognitive Trajectory | Model 1 | P-value | Model 2 | P-value | Model 3 | P-value |
| High-Baseline-Relatively-Stable | 1 [Reference] | | 1 [Reference] | | 1 [Reference] | |
| Low-Baseline-Relatively-Stable | 2.49 (1.66-3.71) | <0.001 | 2.76 (1.79-4.26) | <0.001 | 2.85 (1.84-4.43) | <0.001 |
| Mid-Baseline-Moderate-Decline | 1.88 (1.31-2.72) | <0.001 | 1.78 (1.23-2.57) | 0.002 | 1.85 (1.27-2.68) | 0.001 |


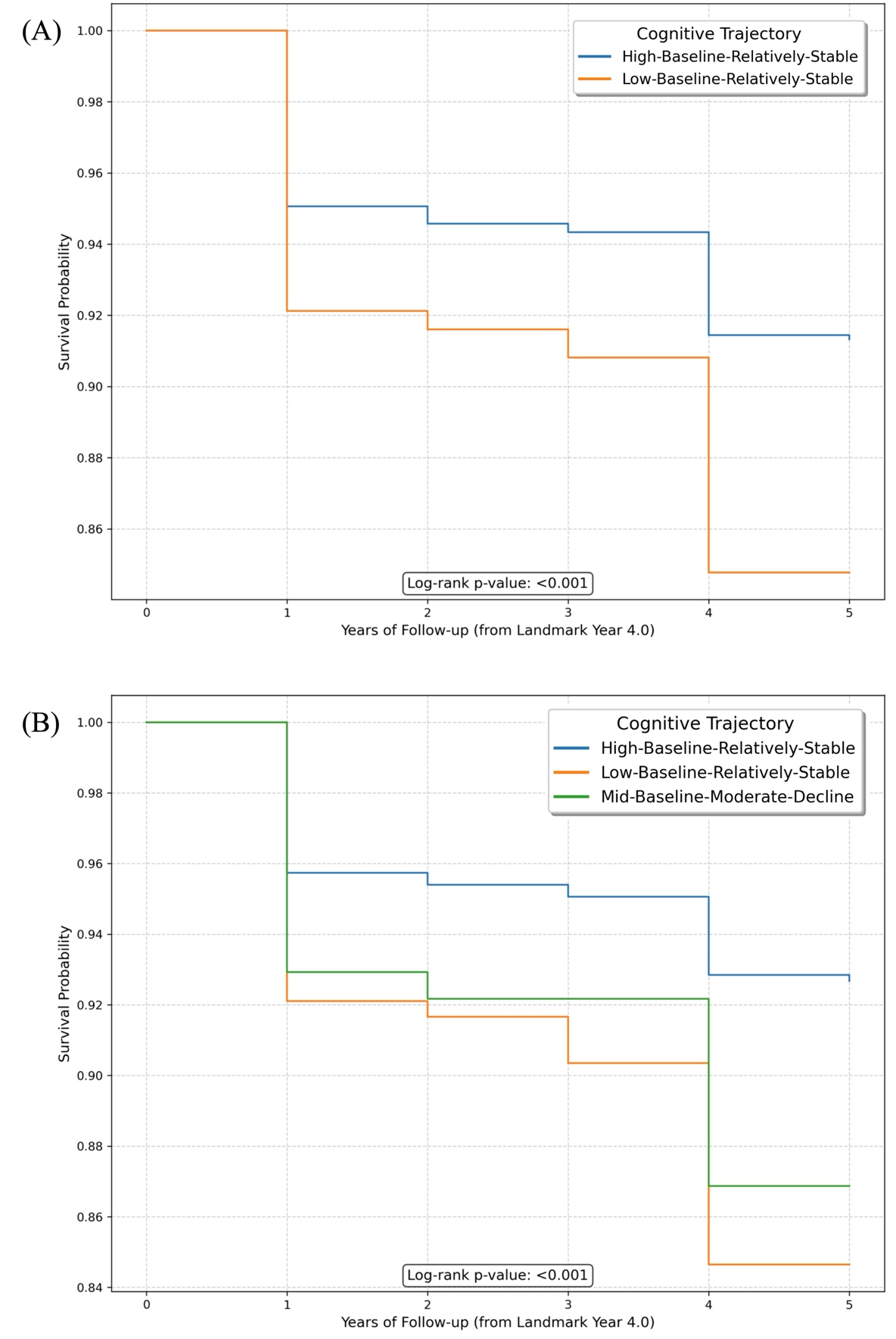


Figure S3. Kaplan-Meier survival curves for all-cause mortality stratified by cognitive trajectory among middle-aged and older Chinese adults with cardiovascular disease in CHARLS (2011-2020). (A) *K=2*;(B) *K*=3. A log-rank test indicated significant differences in survival probabilities among the groups, p < 0.001.

Table S8. Multivariable cox regression models examining the association of cognitive trajectory with all-cause mortality among middle-aged and older Chinese adults with cardiovascular disease in CHARLS 2011-2020 after excluding participants with missing data (N=969, E=106).

| Cognitive Trajectory | Model 1 | Model 2 | Model 3 |
| --- | --- | --- | --- |
| High-Baseline-Relatively-Stable | 0.69 (0.43-1.09) | 0.66 (0.41-1.04) | 0.65 (0.41-1.04) |
| Mid-High-Baseline-Significant-Improvement | 1 [Reference] | 1 [Reference] | 1 [Reference] |
| Mid-Baseline-Rapid-Decline | 1.71 (1.07-2.71) | 1.68 (1.05-2.68) | 1.71 (1.07-2.74) |
| Low-Baseline-Relatively-Stable | 1.90 (1.11-3.27) | 2.09 (1.17-3.73) | 2.04 (1.11-3.73) |

Abbreviations: CVD, cardiovascular disease; CHARLS, the China Health and Retirement Longitudinal Study.

Model 1 was adjusted for none.

Model 2 was adjusted for age, sex.

Model 3 was adjusted for age, sex, BMI, drinking status, smoking status, hypertension, dyslipidemia, and diabetes.

Table S9. Multivariable cox regression models examining the association of cognitive trajectory with all-cause mortality among middle-aged and older Chinese adults with cardiovascular disease in CHARLS 2011-2020 after excluding participants dead within 2 years of follow-up.

| Cognitive Trajectory | Model 1 | Model 2 | Model 3 |
| --- | --- | --- | --- |
| High-Baseline-Relatively-Stable | 0.83 (0.36-1.45) | 0.85 (0.35-1.45) | 0.85 (0.35-1.47) |
| Mid-High-Baseline-Significant-Improvement | 1 [Reference] | 1 [Reference] | 1 [Reference] |
| Low-Baseline-Relatively-Stable | 1.63 (1.15-2.63) | 1.70 (1.10-2.90) | 1.75 (1.12-3.12) |
| Mid-Baseline-Rapid-Decline | 2.01 (1.40-3.08) | 1.95 (1.25-2.85) | 1.97 (1.33-3.02) |

Abbreviations: CVD, cardiovascular disease; CHARLS, the China Health and Retirement Longitudinal Study.

Model 1 was adjusted for none.

Model 2 was adjusted for age, sex.

Model 3 was further adjusted for age, sex, BMI, drinking status, smoking status, hypertension, dyslipidemia, and diabetes.

Table S10. Multivariable cox regression models examining the association of cognitive trajectory with all-cause mortality among middle-aged and older Chinese adults with cardiovascular disease in CHARLS 2011-2020 with additional adjustment for biomarkers and frailty index.

| Cognitive Trajectory | Model 1 | Model 2 | Model 3 |
| --- | --- | --- | --- |
| High-Baseline-Relatively-Stable | 0.74 (0.48-1.13) | 0.76 (0.49-1.16) | 0.77 (0.50-1.19) |
| Mid-High-Baseline-Significant-Improvement | 1 [Reference] | 1 [Reference] | 1 [Reference] |
| Low-Baseline-Relatively-Stable | 1.82 (1.10-3.00) | 1.88 (1.13-3.10) | 1.79 (1.09-2.96) |
| Mid-Baseline-Rapid-Decline | 2.01 (1.32-3.05) | 2.10 (1.37-3.20) | 1.97 (1.29-3.00) |

Abbreviations: CVD, cardiovascular disease; CHARLS, the China Health and Retirement Longitudinal Study.

Model 1 was adjusted for age, sex, BMI, drinking status, smoking status, hypertension, dyslipidemia, and diabetes.

Model 2 was further adjusted for all covariates in Model 3 plus the following biomarkers (after screening for multicollinearity): white blood cell count, mean corpuscular volume (MCV), blood urea nitrogen (BUN), glucose, creatinine, HDL-cholesterol, LDL-cholesterol, c-reactive protein (CRP), and uric acid.

Model 3 further adjusted for all covariates in Model 1 plus frailty index.

Table S11. Baseline characteristics of middle-aged and older Chinese adults with cardiovascular disease by with or without complete cognitive data in CHARLS 2011-2020.

| Characteristic | Overall | Complete Cognitive Data | Incomplete Cognitive Data | P-Value |
| --- | --- | --- | --- | --- |
| Age, mean (SD) | 62.2 (10.2) | 60.3 (9.1) | 64.8 (11.1) | <0.001 |
| Sex, n (%) |  |  |  | 0.99 |
| Male | 1057 (42.2) | 614 (41.9) | 443 (42.7) |  |
| Current Drinker, n (%) | 587 (23.5) | 362 (24.7) | 225 (21.7) | 0.102 |
| Current Smoker, n (%) | 930 (37.2) | 551 (37.6) | 379 (36.5) | 0.673 |
| Hypertension, n (%) | 1332 (53.2) | 744 (50.8) | 588 (56.6) | 0.005 |
| Dyslipidemia, n (%) | 570 (22.8) | 351 (24.0) | 219 (21.1) | 0.107 |
| Diabetes, n (%) | 345 (13.8) | 184 (12.6) | 161 (15.5) | 0.035 |
| BMI, mean (SD) | 24.4 (4.3) | 24.6 (4.3) | 23.9 (4.5) | <0.001 |

Abbreviations: CVD, cardiovascular disease; CHARLS, the China Health and Retirement Longitudinal Study.
